# Effect of Modification of Amorphous Silica with Ammonium Agents on the Physicochemical Properties and Hydrogenation Activity of Ir/SiO_2_ Catalysts

**DOI:** 10.3390/ma14040968

**Published:** 2021-02-18

**Authors:** Monika Kot, Robert Wojcieszak, Ewa Janiszewska, Mariusz Pietrowski, Michał Zieliński

**Affiliations:** 1 Faculty of Chemistry, Adam Mickiewicz University in Poznań, Uniwersytetu Poznańskiego 8, 61-614 Poznań, Poland; monika.kot@amu.edu.pl (M.K.); eszym@amu.edu.pl (E.J.); mariop@amu.edu.pl (M.P.); 2 Univ. Lille, CNRS, Centrale Lille, Univ. Artois, UMR 8181-UCCS-Unité de Catalyse et Chimie du Solide, F-59000 Lille, France; robert.wojcieszak@univ-lille.fr

**Keywords:** commercial silica, modified silica, iridium catalysts, hydrogenation reaction

## Abstract

The modification of commercial silica with solutions of NH_4_F or NH_4_Cl salts, followed by thermal treatment, enabled generation of the acidic sites in SiO_2_ and changed its textural properties. The use of ammonium salts solution also caused the generation of additional porosity. Using NH_4_F solution caused significant decrease in the specific surface area and the increase in the average pore diameter. The number and strength of resulting acid sites depend on the nature of anion in the applied ammonium salt and the concentration of salt solution. It has been found that the sample treated with NH_4_F presented higher total acidity (TPD–NH_3_) and the amount as well as the strength of acid sites increased with the concentration of the used modifier. As modified amorphous SiO_2_ materials used as a support for iridium (1 wt %, Ir(acac)_3_) nanoparticles permitted to obtain highly active catalysts for toluene hydrogenation under atmospheric pressure. The highest activity (expressed as the apparent rate and TOF) was obtained for iridium catalysts supported on silica modified by NH_4_F with the highest acidity. The modification of silica with NH_4_F favors the generation of centers able to adsorb toluene, which results in higher activity of this catalyst.

## 1. Introduction

Hydrogenation reactions have become industrially important since 1897, when Sabatier found out that the application of nickel as a catalyst facilitates the addition of hydrogen to molecules of gaseous hydrocarbons [1]. Nowadays, hydrogenation processes still attract considerable attention. For example, benzene hydrogenation leads to obtaining cyclohexane used in the production of nylon fibers and resins [2,3].

The catalytic activity in the processes of hydrogenation depends on both the type of active phase and properties of the support used. The surface properties of support, its crystalline and porous structure affect the dispersion of active phase and determine its reducibility. For that reason, the fundamental task of heterogeneous catalysis is to find the most beneficial combination of metal active phase and support. The choice of catalyst requires many variables to be taken into account, whereas the type of catalytic process seems to be key factor.

It has been demonstrated that the support used to disperse an active phase can affect catalyst activity for aromatic hydrocarbons hydrogenation. Several authors have evidenced that the performance of metal catalysts supported on acidic supports for hydrogenation of different organic compounds—e.g., benzene, toluene, and xylene—is enhanced in comparison to catalysts on inert supports, e.g., SiO_2_ [4,5,6,7,8] and ordered mesoporous silica [9,10,11]. It was found that aromatics adsorbed on acid site can be hydrogenated with the activated hydrogen spillover from metal centers [4,5]. These results clearly indicate that the acidity of support is crucial for catalytic activity. Zeolites, as solid acids, are commonly used in catalysis. Unfortunately, their high acidity facilitates side reactions (cracking, alkylation, isomerization) and coke formation, leading to deactivation of catalyst [12]. No decrease in activity was observed when the active phase was deposited on the supports with weak and intermediate acid sites [13].

Amorphous silica materials are the most significant class of catalyst supports due to low price, hardness, chemical resistance, thermal stability, and non-toxicity. However, pure silica materials show no acidity or only low acidity. The amorphous as well as the crystalline silica (e.g., silicalite-1 with MFI structure) possess a very weak acidity due to their silanol groups. The acidity of silica depends on the amount of the silanol groups and their form (acidic silanol nests or vicinal silanols, non-acidic isolated silanols), which in turn depends on the method of its preparation or the post-synthesis treatment (e.g., calcination) [14]. The acidic properties of mesoporous silica can be modified by isomorphous substitution (e.g., obtaining AlMCM-41 [15] or AlSBA-3 [9,16]) or using post-synthesis method [17]. Some studies have also indicated that the treatment of siliceous counterparts of zeolites, particularly of MFI-type (silicalite-1), with aqueous solution of different ammonium salts and basic compound—e.g., aqueous ammonia solution, alkylamines, or allylamines—resulted in enhanced activity in the Beckmann rearrangement reaction (an acid-catalyzed rearrangement of an oxime to an amide) [18,19,20]. In addition, MFI-type silicalite modified with ammonium agents have been also applied for etherification of HMF [21] and acetalization of glycerol with acetone [22]. It has been shown that amorphous silica modified with aqueous solution of ammonium chloride or ammonium nitrate in presence of ammonia solution generates weak acidic properties of the material. The results presented in the literature confirmed that the presence of acid sites is essential for toluene hydrogenation [8]. The modification of silica materials using ammonium agents may be a simple, cheap, and effective route to obtain catalysts with enhanced activity for hydrogenation reactions. The modification procedure is simple and does not require multiple steps. Moreover, it involves application of nontoxic low-cost ammonium salts. For this reason, the modified supports may have potential for application in industrial-scale process.

Janiszewska et al. [22] indicated that the number and strength of acid sites generated in MFI-type silicalite by treatment with aqueous solution of ammonium salts depend on the nature of anion in the applied ammonium salt. The highest overall acidity and the higher contribution of stronger acid centers have been indicated by samples modified with aqueous solution of NH_4_Cl and NH_4_F. Additionally, the modification of silicalite-1 by NH_4_Cl or NH_4_F solutions caused formation of additional mesoporosity which resulted in the improvement of catalytic activity through better availability of acid centers [22]. On the other hand, in the case of cobalt catalysts for Fischer–Tropsch synthesis, silica was modified with ammonium nitrate in order to reduce surface hydroxyls resulting in weaken metal-support interaction [23], whereas the pretreatment of silica with ammonia solution led to stronger interaction between cobalt and silica [24]. Both strategies improved catalytic activities of investigated samples.

The aim of this work was to modify commercial amorphous silica using ammonium agent solutions in order to generate acid centers in starting material. The influence of the ammonium salts used (NH_4_Cl or NH_4_F) and its concentration (0.1 M or 1.0 M) on the acidity and textural properties of the obtained amorphous silica was investigated. It was the first attempt to use NH_4_F as a modifier of amorphous silica to generate its acidic properties. The obtained silica materials were used as supports for iridium catalysts. The effect of support acidity on the activity of catalysts for toluene hydrogenation was studied.

## 2. Materials and Methods

### 2.1. Supports and Catalysts Preparation and Activation

Amorphous silica (Polish Chemical Reagents, Gliwice, Poland) was modified with 0.1 and 1.0 M solutions of ammonium chloride (NH_4_Cl—Aldrich, Saint Louis, Missouri, USA) and ammonium fluoride (NH_4_F—Polish Chemical Reagents, Gliwice, Poland). The samples of silica were calcined before modification for 3 h at 550 °C. A portion of silica (1 g) was mixed with 100 cm^3^ of aqueous solution of NH_4_Cl or NH_4_F. The obtained mixture was stirred under reflux at temperature 60 °C for 1 h. After the treatment, the silica modified materials were filtered, washed, and dried at RT (room temperature). Finally, the samples were calcined in the muffle furnace in the air (550 °C; 3 h). The resulting samples were labeled as SiO-F-x (sample modified with solution of ammonium fluoride) and SiO-Cl-x (sample modified with solution of ammonium chloride) where x means concentration of modifying agent. For comparison, the unmodified silica, labeled as SiO was also used as a support.

Iridium was deposited on silica supports by conventional impregnation method using iridium acetylacetonate (Ir(acac)_3_—Aldrich, Saint Louis, MO, USA) as a metal precursor. The metal precursor was dissolved in methanol (Polish Chemical Reagents, Gliwice, Poland) and the calcined support was added to the iridium solution (the amount of iridium precursor was calculated to achieve metal loading of 1 wt %). After stirring the solvent was removed using rotary evaporator and the catalysts were dried (105 °C; 24 h). The obtained materials were labeled as Ir-SiO, Ir-SiO-Cl-0.1, Ir-SiO-F-0.1.

Prior to the measurements of hydrogen chemisorption, as well as before the measurements by low-temperature N_2_ adsorption/desorption (BET), X-ray photoelectron spectroscopy (XPS), X-ray powder diffraction (XRD), X-ray fluorescence (XRF) and Fourier-transform infrared spectroscopy (FTIR) measurements, each precursor-impregnated support was placed in a fixed-bed flow reactor and reduced in hydrogen flow (Linde, 50 cm^3^ min^−1^, 400 °C, 2 h). Only for temperature-programmed reduction with hydrogen (TPR-H_2_) measurement, dried precursors of catalysts were used.

### 2.2. Characterization of Supports and Catalysts

The Brunauer-Emmet-Teller (BET,) surface areas (S_BET_) were determined by low-temperature (−196 °C) nitrogen adsorption/desorption using a Micromeritics model ASAP 2010 sorptometer (Micromeritics, Norcross, GA, USA). Total pore volume and average pore diameter were determined by the Barrett–Joyner–Halenda (BJH) method.

The temperature-programmed desorption of ammonia measurements (NH_3_-TPD) were carried out on Micromeritics (Norcross, GA, USA) model Pulse ChemiSorb 2705 instrument. A portion of the support (~500 mg) was activated in-situ for 1 h in He (Linde) at the rate of 10 °C min^−1^ up to 450 °C. Next it was cooled to 100 °C and contacted with ammonia for 10 min at a flow rate of 10 cm^3^·min^−1^ followed by flushing with helium (30 cm^3^·min^−1^; 1 h). Then, the NH_3_-TPD analysis was performed in the temperature range of 100–500 °C with a heating rate of 10 °C min^−1^ using TCD detector. The NH_3_-TPD profiles were normalized to the same sample weight (1 g). The calibration was performed with a calibrated gas loop by dispensing 1 cm^3^ NH_3_ into the helium flow.

FTIR analysis was performed using FTS 3000 Bio-Rad (Bio-Rad, CA, USA) spectrophotometer connected to a conventional vacuum system. Detailed experimental procedure of FTIR analysis is presented in Supplementary Materials. Pyridine adsorption (6.0 mbar) was carried out at 50 °C for 10 min. Spectra were recorded after evacuation at 50, 75 and 100 °C. Toluene adsorption (5.0 mbar) was carried out at room temperature for 10 min. Spectra were recorded after evacuation at RT.

XRD analysis was performed in the 2Θ range between 10° and 60° on a Bruker AXS D8 Advance (Billerica, MA, USA) diffractometer with Ni-filtered CuK α radiation (λ = 1.54056 Å).

Iridium content in catalysts after activation was determined by XRF measurements using energy dispersive micro X-ray fluorescence spectrometer M4 TORNADO (Bruker, Billerica, MA, USA).

Measurements of TPR-H_2_ were carried out on Pulse ChemiSorb 2705 (Micromeritics, Norcross, GA, USA) instrument. Dried metal precursor-impregnated supports (~50 mg) were reduced in the flow of 10 vol %. H_2_-Ar (Linde) at the flow rate of 30 cm^3^ min^−1^ in the temperature range from 50 to 700 °C with the heating rate of 10 °C min^−1^. In the TPR-H_2_ studies, a quartz sand (Aldrich) impregnated with iridium acetylacetonate was used as a reference material. All TPR-H_2_ profiles have been normalized to the same sample weight.

Chemisorption of H_2_ experiments were performed on an ASAP 2010C (Micromeritics, Norcross, GA, USA) sorptometer. Detailed experimental procedure of H_2_ chemisorption analysis is presented in Supplementary Materials.

Iridium surface area (*S,* m^2^ g_Ir_^−1^*)* was calculated using the following equation [25]

(1) $$S=\frac{v_{m}\cdot N_{A}\cdot n\cdot a_{m}\cdot 100}{22,414\cdot m\cdot wt}$$

where *v_m_*—volume of adsorbed hydrogen (cm^3^); *N_A_* is Avogadro’s number (6.022 × 10^23^ mol^−1^); *n*—chemisorption stoichiometry (*n* = 2); *a_m_*—surface area (m^2^) occupied by an iridium atom; *m*—mass of sample (g); *wt* (%)—iridium loading.

The dispersion *(D)* of iridium phase was calculated according to the formula

(2) $$D=\frac{S\cdot M}{a_{m}\cdot N_{A}}$$

where *S*—the iridium surface area; *M*—iridium atomic weight; *N_A_*—Avogadro’s number; and *a_m_*—surface covered by one iridium atom. The metal content determined by X-ray fluorescence measurements was taken in the calculation of the dispersion.

The average iridium particle size (*P*, nm) was calculated using the following equation

(3) $$P=\frac{6000}{S\cdot \rho }$$

where *S*—the iridium surface area; ρ—metal density (g·cm^−3^).

X-ray photoelectron spectroscopy (XPS) analysis of the iridium catalysts was carried out with a Kratos Axis Ultra spectrometer (Kratos Analytical, Manchester, UK). The excitation source was monochromatized aluminum X-ray source (Al Kα (1486.6 eV)) operated at 10 mA and 15 kV. The spectra were referenced to the binding energy of C (1 s) (284.6 eV). Spectroscopic data were processed by the CasaXPS ver. 2.3.17PR1.1 software (Casa Software Ltd., Teignmouth, UK), using a peak-fitting routine with Shirley background.

Toluene hydrogenation was performed at atmospheric pressure using a fixed-bed flow reactor and H_2_ as a carrier gas. The scheme (Figure S1) of the setup used for toluene hydrogenation and detailed experimental procedure are presented in Supplementary Materials.

The catalytic activity is presented as turnover frequency (TOF, min^−1^—in moles of toluene reacted per surface iridium atoms for 1 min) or as apparent rate (*r_t_*) calculated according to the following equation [26]

(4) $$r_{t}=\;\frac{FYC}{N}$$

where *F*—total flow rate (cm^3^ min^−1^); *Y*—conversion of toluene; *C*—concentration of toluene in the feed (mol_Tl_ cm^3^); and *N*—iridium content (mol_Ir_) in the sample. Turnover frequency (abbreviation TOF) was calculated by dividing the number of toluene molecules converted per unit time by the number of active iridium atoms.

## 3. Results and Discussion

### 3.1. Characterization of the Modified Amorphous Silica

The amorphous silica was modified with the use of ammonium salts solutions in order to generate acid centers on its surface. For this purpose, NH_4_Cl and NH_4_F solutions with concentrations of 0.1 and 1.0 M were used. The modified silica materials obtained in the work are new systems that have never been used as supports in catalysis. Hence, the characteristics of the porosity and the specific surface are a very important parameter characterizing new supports. Based on low-temperature nitrogen adsorption–desorption measurements, the textural properties of unmodified and modified silicas were determined. The obtained samples contained mesopores in the size range of 3 to 13 nm, while the BET surface area changed from 311 to 117 m^2^·g^−1^ (Table 1).

**Figure 1:**
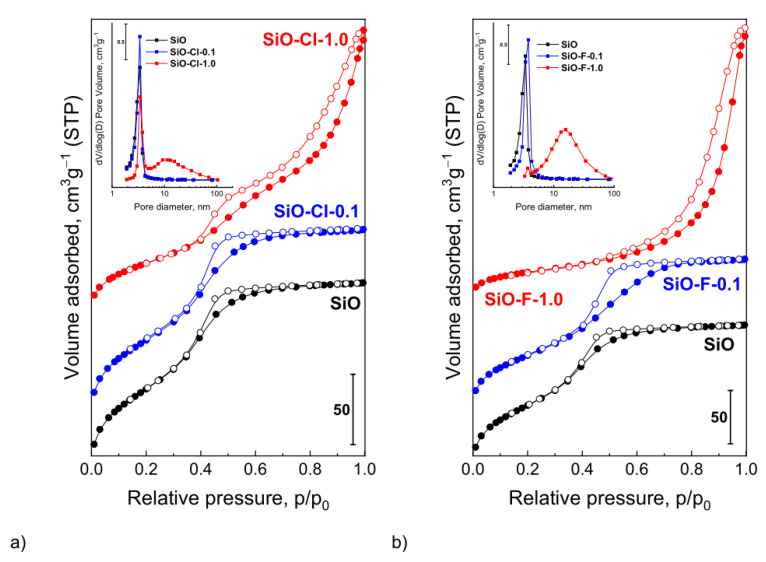
Low-temperature N_2_ adsorption/desorption isotherms and pore size distribution (inset) of unmodified silica and silica samples modified by NH_4_Cl (**a**) and NH_4_F (**b**).

The studies of low-temperature N_2_ adsorption indicate that the use of the modifiers with a concentration of 1.0 M caused generation of additional porosity—the value of the S_BET_ drops from 311 m^2^∙g^−1^ for the initial silica to 183 m^2^∙g^−1^ for the SiO-Cl-1.0 sample and 117 m^2^∙g^−1^ for SiO-F-1.0 with a simultaneous increase in the average pore diameter to 6.5 and 13 nm for SiO-Cl-1.0 and SiO-F-1.0, respectively. The obtained results also show that the use of NH_4_F solution causes a higher decrease of the S_BET_ and an over fourfold increase in the average pore diameter in relation to the unmodified silica. The use of modifiers with a lower concentration (0.1 M) does not lead to such drastic changes both in the S_BET_ and in the porous properties. The textural changes are particularly seen in the shape of the N_2_ adsorption–desorption isotherms—Figure 1.

The initial SiO_2_, according to the new IUPAC classification, is characterized by type IV(a) isotherm and H2(a) hysteresis loop [27]. The shape of isotherm is characteristic of mesoporous materials having a pore system for which network effects play a significant role. The very steep desorption branch, which is a characteristic feature of H2(a) loops, can be attributed either to pore-blocking/percolation in a narrow range of pore necks or to cavitation-induced evaporation. SiO material is characterized by a very narrow pore size distribution, mainly in the range of 2–4 nm (with average pore size of 3.0 nm). The initial silica and samples after modification with the 0.1 M ammonium solutions show similar adsorption isotherms. The slightly larger hysteresis loop for samples modified with 0.1 M solutions in comparison to the initial silica is due to the formation of new mesopores during the modification process. The position of hysteresis loop in the same range of p/p_0_ = 0.4–0.7 indicates the similar pore sizes of the initial and modified samples that is consistent with pore size distribution profiles (Figure 1, inset). The shape of the N_2_ adsorption–desorption isotherms change after using ammonium salts with a concentration of 1.0 M. The desorption isotherm of SiO-Cl-1.0 sample has an inflection in the relative pressure p/p_0_ = 0.4–1.0 indicating the presence of pores of different sizes. The pore size distribution curve (Figure 1a, inset) shows that apart the pores with a size of ~3 nm, there are additional pores with a size of ~10 nm. Such an inflection was not recorded for the SiO-F-1.0 system, the hysteresis loop is shifted to higher p/p_0_, and the pore size distribution was in the range of 3–50 nm with a maximum of ~13 nm—Figure 1b. For systems modified with 1.0 M solutions, all isotherms are of type IV(a) and hysteresis loops of type H2(b). The presence of hysteresis loop of type H2(b) and parallel adsorption and desorption branches (p/p_0_ 0.7–0.9) indicates that the pores of obtained materials have a cylindrical shape, while the fact that the loop closes at p/p_0_ = 0.4 suggests the presence of small cone-shaped pores.

**Figure 2:**
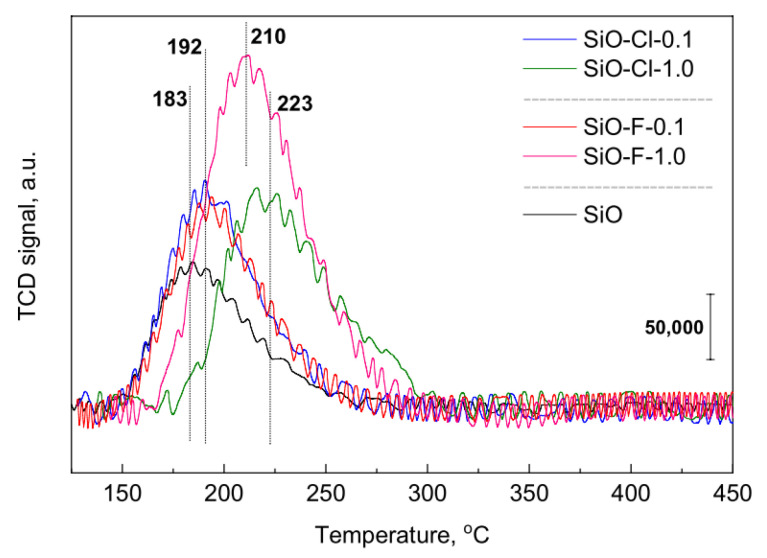
NH_3_-TPD profiles of silica samples calcined at 550 °C.

NH_3_-TPD analysis was performed to characterize acidic properties of samples, assess the acid strength distribution, and determine the concentration of acid sites. The NH_3_-TPD profiles are presented in Figure 2 and results of their deconvolution in Figure S2.

**Figure 3:**
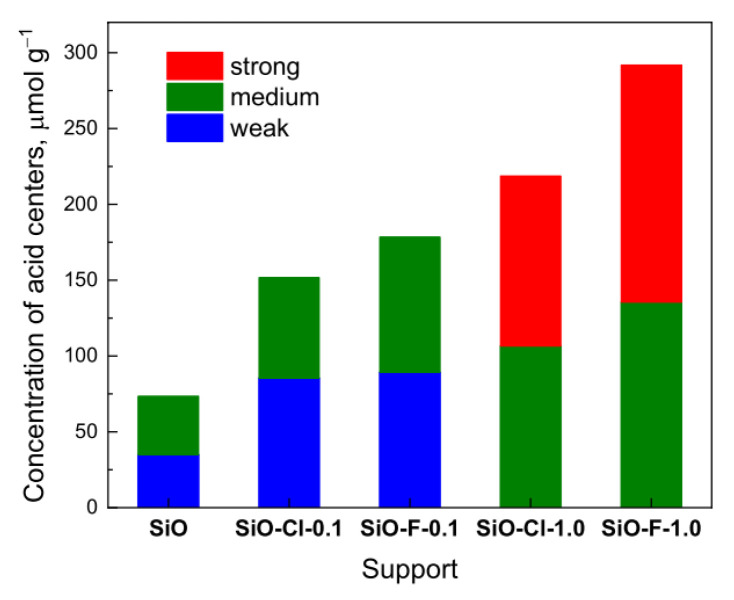
Concentration of different types of acid centers on the surface of indicated silica materials calculated from NH_3_-TPD studies.

Based on the shape of the NH_3_-TPD profiles it can be concluded that the investigated samples contain acid centers of different strengths. The traditional classification [28] describes the existence of two kinds of acid centers: weak acid centers assigned to low-temperature peaks (below 400 °C) and strong acid centers ascribed to high-temperature peaks (above 400 °C). The low-temperature peaks refer to desorption of ammonia from weakly acidic silanol groups and weak Lewis acid centers, while high-temperature peaks correspond to desorption of ammonia from strong Brønsted and Lewis acid sites [28]. The NH_3_-TPD profiles in the case of our samples indicate only desorption peaks below 400 °C attributed to weak acid sites. Due to the fact that the desorption maxima for all samples were recorded at different temperature, the deconvolution of the peaks was applied. Moreover, a new specific classification of the strength of acid sites, that will be more suitable for presented silica materials, was introduced. The desorption peak at about 180 °C can be assigned to weak acid sites, whereas peaks at and above 200 °C refer to medium strength and strong acid sites, respectively. The concentration of surface acid centers was calculated (Table 1) on the basis of the NH_3_-TPD profiles and after deconvolution of the profiles (Figure S2), the concentration of the weak, medium, and strong acid centers was evaluated (Figure 3). The modification of pristine support causes the formation of additional acid centers on its surface. The number and the strength of formed acid sites depends on the type and concentration of ammonium agent applied for a modification. The initial silica (SiO) shows a slight acidity (73.5 µmol∙g^−1^) and the modification of its surface with 0.1 M solutions leads to the generation of additional acid centers, especially of low and medium strength. In the case of samples modified with 1.0 M solutions, an increase in the concentration of strong acid centers is observed with a simultaneous decrease in the contribution of weak acid centers—Figure 3.

**Figure 4:**
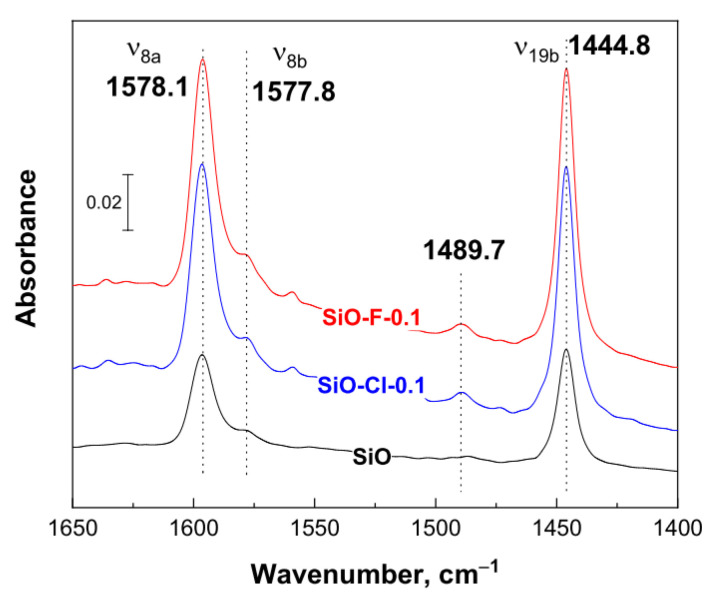
FTIR spectra of pyridine adsorbed on the modified and unmodified SiO_2_ after evacuation at 50 °C.

Based on TPD-NH_3_ the strength and concentration of acidic centers on the surface of investigated silica materials were determined whereas the nature of acid sites was characterized using FTIR spectroscopy using pyridine as a probe molecule. In Figure 4, FTIR spectra recorded after pyridine adsorption on the surface of pristine and selected modified silica supports are presented.

In the spectra of the starting silica as well as for materials modified with 0.1 M solutions of ammonium salts bands characteristic of pyridine coordinately bounded to Lewis acid centers were observed: ν_19b_ at 1444.8 and ν_8a_ at 1578.1 cm^−1^. No presence of Brønsted acid centers (ν_19b_ at 1532 cm^−1^ and ν_8a_ at 1643 cm^−1^) was found [29]. The band at 1444.8 cm^−1^ can also originate from Py hydrogen-bounded to surface hydroxyl or silanol groups [30]. A band at 1578.1 cm^−1^ corresponds to pyridine bounded to weak Lewis acid sites [31]. Weak band at 1489.7 cm^−1^, seen only for the samples after modification, is associated with both Lewis and Brønsted acid sites [31]. Because of the lack of a band at 1532 cm^−1^, it could be concluded that the band at 1489.7 cm^−1^ originates only from Lewis acid centers. The same bands are presented in the spectra of samples modified with 1.0 M solutions of ammonium salts (spectra not shown).

Summarizing the results of the research on the modification of amorphous silica with the use of ammonium salts of various concentrations, it can be concluded that the modification with NH_4_Cl and NH_4_F solutions with concentrations of 0.1 M allows to obtain mesoporous supports with a surface area comparable to the original silica, and their modification leads to the generation on their surface of the Lewis type acid centers of weak and medium strength. Hence, in the further part of the research, these systems were selected as supports for the iridium active phase.

### 3.2. Iridium Catalysts

Selected supports were used to prepare iridium catalysts. The precursor of the active phase was iridium acetylacetonate (Ir(acac)_3_) and metal loading was 1 wt %. The X-ray fluorescence (XRF) measurements of all the iridium catalysts have indicated the similar amount of iridium but lower amount than the intended ones—Table 2. The bare silica showed the greatest difference in the amount of iridium in relation to the intended amount. In the further part of the research (dispersion of the active phase and studies of hydrogenation activity), the iridium content determined on the basis of XRF was taken into consideration.

**Figure 5:**
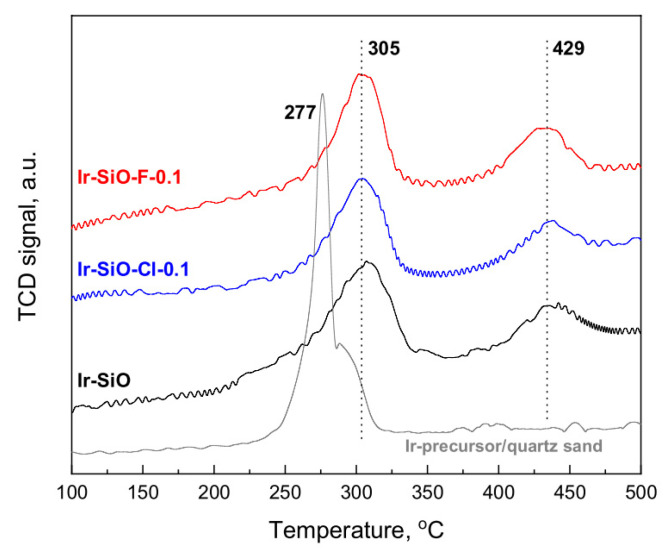
Temperature programmed reduction profiles of the dried catalysts and iridium precursor (Ir(acac)_3_). Signal intensity was standardized to the same samples weight (100 mg).

Temperature programmed reduction with hydrogen (TPR-H_2_) was used to determine the temperature needed to decompose iridium precursor ligands with hydrogen and remove them from the catalyst surface. TPR-H_2_ studies were carried out on dried samples and reduction profiles of fresh catalysts precursors dried at 105 °C are presented in Figure 5.

The TPR-H_2_ profile of iridium precursor (Ir(acac)_3_) indicates one peak with maximum at the temperature 277 °C with small shoulders at ~300 °C. However, Ir(acac)_3_ deposited on modified and unmodified silica materials shows hydrogen consumption with two separated maxima at ~300 °C and ~430 °C, which indicates two-steps decomposition and reduction of active phase precursor. No hydrogen consumption was seen in profiles of the supports (data not presented), which, as expected, proves that the supports were irreducible. According to the authors of [32], thermal decomposition of acac ligands in Ir-acac_x_ species took place at a temperature of about 320 °C. It indicates that the peak at ~300 °C in the presented profiles originates from the decomposition of acac ligands. The peak with the maximum at 380–400 °C, visible in presented profiles could be attributed to a reductive decomposition of Ir-acac_x_ species [32]. The deconvolution of the reduction profiles of investigated samples in the 200–400 °C range (Figure S3) indicates additionally presence of iridium oxide particles on the catalyst surface with the maximum reduction at ~275 °C. As reported by the authors [33], this peak can be identified as well-dispersed iridium species. The presence of iridium oxide particles is probably due to the degradation of part of Ir (acac)_3_ to IrO_2_ during drying of the catalysts. The TPR-H_2_ results showed that relatively high temperatures are needed for reductive decomposition of acac ligands in iridium catalysts supported on modified and unmodified silicas. However, the reduction temperature of 400 °C used in the work is sufficient for the decomposition and reduction of the metallic phase precursor. In contrast to the TPR-H_2_ measurements, the catalyst activation (Section 2.1) was carried out in pure hydrogen and in extended time (2 h), which allowed the complete transition of the active phase precursor to the metallic iridium. In addition, the carried out TPR-H_2_ measurements of the reduced catalysts showed no reduction peaks indicating the presence of non-reduced active phase.

Measurements of low-temperature nitrogen adsorption/desorption were carried out for catalysts reduced at 400 °C. Table 2 shows the specific surface area, total pore volume, and average pore diameter of the catalysts investigated. The N_2_ adsorption/desorption isotherms of iridium catalysts strongly resemble those obtained for supports, therefore the isotherms of catalysts are shown in Supplementary Material (Figure S4).

**Figure 6:**
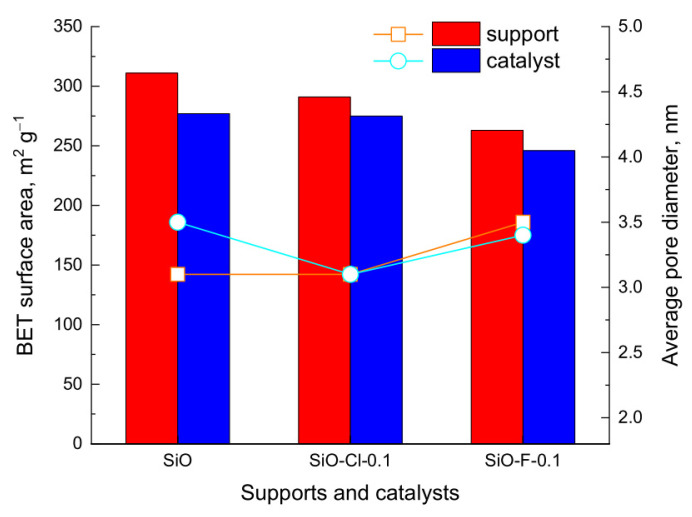
BET surface areas (bars) and average pore diameters (dots) of supports (red) and iridium catalysts (blue).

The low-temperature nitrogen adsorption–desorption isotherms were used to calculate surface area of supports and iridium catalysts—Figure 6. As already mentioned in the discussion of the results of N_2_ adsorption–desorption measurements of supports (Section 3.1) the S_BET_ of supports decreases from 311 m^2^∙g^−1^ for unmodified silica to 263 m^2^∙g^−1^ for SiO-F-0.1. A similar tendency is observed in the case of the surface area of catalysts. However, the catalysts show always somewhat lower surface area than the surface area of relevant supports. The decrease in surface area of the catalysts is a result of the deposition of active phase and additional thermal and wet treatment during catalyst preparation. The impregnation of the supports and the subsequent thermal treatment of the catalysts also affect the average pore diameter, which is particularly seen for unmodified silica—Figure 6. For the latter sample, the largest decrease in BET surface area as well as some increase in average pore diameter was observed.

**Figure 7:**
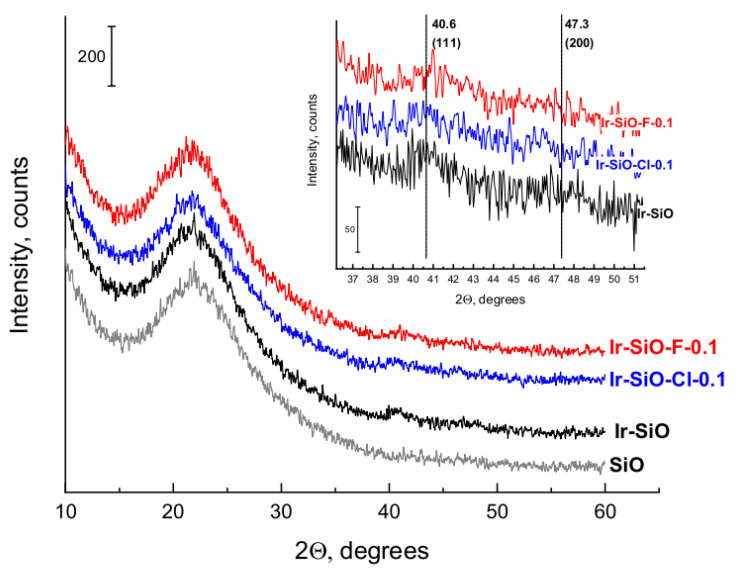
X-ray diffraction patterns of unmodified silica and iridium catalysts reduced at 400 °C.

In Figure 7 are presented X-ray patterns of unmodified silica and iridium catalysts. The wide reflection in the 2Θ range of 15–30° observed for all materials is ascribed to amorphous silica.

The XRD patterns of iridium catalysts show weak reflections at 2Θ = 40.6° (111) and 47.3° (200) corresponding to metallic iridium with a fcc structure [34]. The intensity of the reflections is very small, although noticeable, which for such a low loading of the support surface may indicate a crystallite size of about 5 nm. Particularly visible is the reflex at 2Θ = 40.6° for Ir-SiO catalyst. It indicates that the modification of the silica support leads to a better dispersion of active phase for Ir-SiO-Cl-0.1 and Ir-SiO-F-0.1 catalysts. The XRD results were confirmed by hydrogen chemisorption measurements.

As the iridium loading was very low (0.7–0.8 wt %), therefore confirmation of Ir^0^ phase by XRD analysis was difficult. For this reason, a suitable method that provides detailed information about iridium particle size can be hydrogen chemisorption measurements. The experiments were performed in a volumetric system at 35 °C. Before the measurements, each catalyst was activated in-situ at 360 °C in H_2_ flow. On the basis of hydrogen being adsorbed irreversibly, calculations of average particle size and dispersion of active phase were performed. Results of the calculations are presented in Table 3.

The results of hydrogen chemisorption measurements show that dispersion depends on the textural properties of the support. The catalysts supported on modified silica with higher surface area show higher dispersion of iridium particles and smaller iridium particles than the catalyst supported on parent silica. The NH_3_-TPD studies for our supports (SiO-F-0.1 and SiO-Cl-0.1—Table 1 and Figure 3) indicated a higher total number of acid centers for SiO-F-0.1 material. The higher acidity of the SiO-F-0.1 surface favors a slightly better dispersion of the iridium active phase. The obtained results are in good agreements with data reported for modified amorphous silica in our previous work [8].

In order to examine the electronic states of the metal functions in iridium catalyst samples, the XPS study was carried out. The binding energy (BE) values for all the catalysts were interpreted after applying the charge correction using C 1s spectra (BE = 284.6 eV).

**Figure 8:**
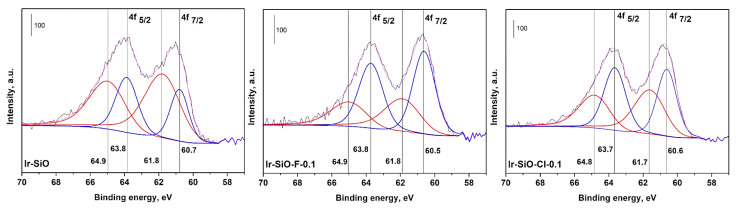
X-ray photoelectron spectra (Ir 4f core levels) for iridium catalysts: Ir-SiO, Ir-SiO-Cl-0.1 and Ir-SiO-F-0.1.

Figure 8 shows the XPS spectra of Ir 4f region of the Ir/silica catalysts. Two doublets with 4f_5/2_ and 4f_7/2_ components are observed originated from the spin orbit coupling of the 4f orbital. The doublet separation is of 3.1 eV which correspond well to the standard values for iridium [35]. The XPS measurements confirmed the presence of two different species on the surface: Ir^0^ and Ir^2+^. The existence of a metallic Ir^0^ on the surface is confirmed by the presence of low energy peaks at around BE = 60.6 eV and 63.7 eV [36]. In addition, the presence of the oxidized form of iridium oxide (IrO_2_) is also observed in all samples (BE of 61.7 and 64.9 eV corresponding respectively to 4f_7/2_ and 4f_5/2_ components) [36,37]. The latter is probably due to the brief air exposure before analysis. Moreover, as could be seen from Table 4, Ir-SiO-F-0.1 catalyst presents much higher extent of the reduced Ir as compared to other samples.

The catalytic performance of iridium samples was evaluated in the reaction of toluene (TL) hydrogenation. Hydrogenation of toluene is a simple reaction with just one hydrogenation product, methylcyclohexane (MCH). When the TL hydrogenation was carried out in the presence of silica supports (SiO, SiO-Cl-0.1, and SiO-F-0.1) no products were observed as a conversion of toluene was null for all supports. This confirms the lack of activity of the supports. Other important parameter that could affect the activity of investigated catalysts is the mass transfer limitations. The Wheeler–Weisz modulus (φ^2^η) evaluated for all iridium sample was lower than 1 within the temperature range used in this work, thus allowing us to neglect diffusional limitations in our experimental conditions [38].

**Figure 9:**
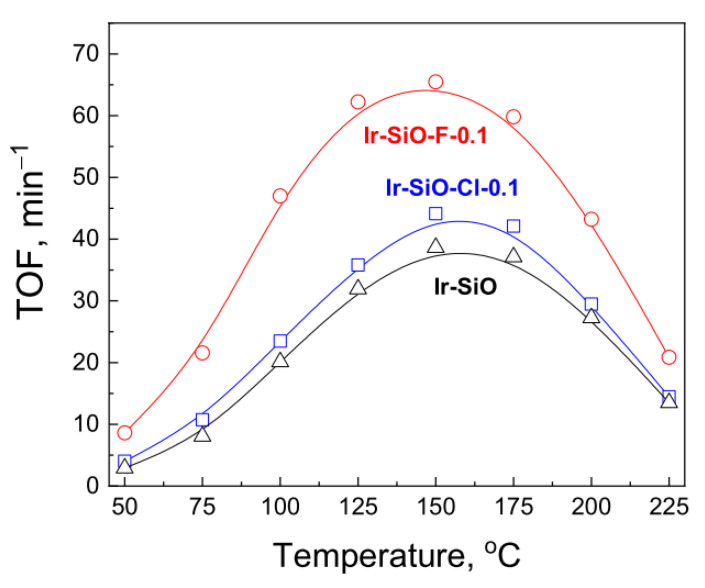
Effect of support on the turnover frequencies of hydrogenation of TL as a function of reaction temperature.

Turnover frequency of toluene hydrogenation (TOF, min^−1^) was calculated based on the number of iridium surface atoms obtained from the volume of chemisorbed hydrogen (H_irr_—Table 3). Turnover frequencies of the investigated catalysts as a function of temperature reaction are shown in Figure 9. Optimum reaction conditions were established on the basis of our earlier results reported in [39].

The best catalytic properties in toluene hydrogenation (highest TOF (Figure 9) and the apparent rate (Table 3)) shows iridium catalyst supported on silica modified by NH_4_F. The activity of iridium catalysts increased with increasing reaction temperature, reaching a maximum at 150 °C for all catalysts. Further temperature increase resulted in a reduction of the activity. The loss of catalytic activity at higher temperatures is due to the occurrence of the dehydrogenation or cracking of MCH formed during the reaction [40]. The other possibility is a decrease in the number of active sites due to poisoning the surface of the catalyst by carbon deposit obtained by cracking of molecules of reactants [40]. However, in the case of iridium catalysts supported on silica modified with ammonium salts, the cracking products were not observed and the only detected product was methylcyclohexane. It clearly indicates that the loss in catalytic activity at higher temperatures was caused by a reverse reaction, i.e., MCH dehydrogenation.

**Figure 10:**
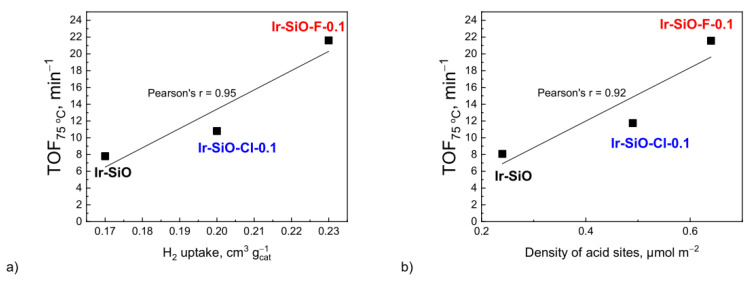
Turnover frequency (TOF) at 75 °C in toluene hydrogenation reaction as a function of total hydrogen uptake (**a**) and density of acid sites (**b**) of iridium catalysts.

The catalytic performance for toluene hydrogenation is affected by the type of support, particularly its textural and acidic properties. The activity of the catalysts increased with the increase of the acidity of the supports. Turnover frequencies at 75 °C as a function of total hydrogen uptake and density of acid sites are shown in Figure 10a,b.

The activity of the catalysts in toluene hydrogenation reaction depended on hydrogen uptake. The best catalytic performance, expressed as TOF at 75 °C was observed for Ir-SiO-F-0.1. As was mentioned before, the high activity of Ir-SiO-F-0.1 may be a result of the higher acidity of the support, which can enhance hydrogenation reaction [41] and the relatively higher hydrogen uptake values, indicating also smaller particle sizes of iridium phase. An increase in TOF value is observed with an increase in hydrogen uptake for the iridium catalysts. Due to the fact that the size of iridium crystallites for Ir-SiO-Cl-0.1 and Ir-SiO-F-0.1 catalysts is similar, hence the difference in TOF is not only due to hydrogen uptake but also to the acidity of the supports—Figure 10b.

As was suggested by the authors [42], the toluene adsorbed on the catalysts surface can be hydrogenated by hydrogen adsorbed through two different mechanisms. Hydrogen adsorbed on the conventional metal active sites and the spillover hydrogen that migrates from the metal sites to the acidic sites. The authors [5,43] reported that Lewis acid sites, being electron-deficient, can easily adsorb aromatic molecules because of their π-bonds are electron donors. This adsorption occurs with the ring parallel to the surface [44]. The electron-deficient aromatic intermediates like the MCH intermediate which form on these sites can be easily hydrogenated. Therefore, in order to explain the differences in activity between Ir-SiO-Cl-0.1 and Ir-SiO-F-0.1 catalysts, we conducted toluene adsorption studies.

**Figure 11:**
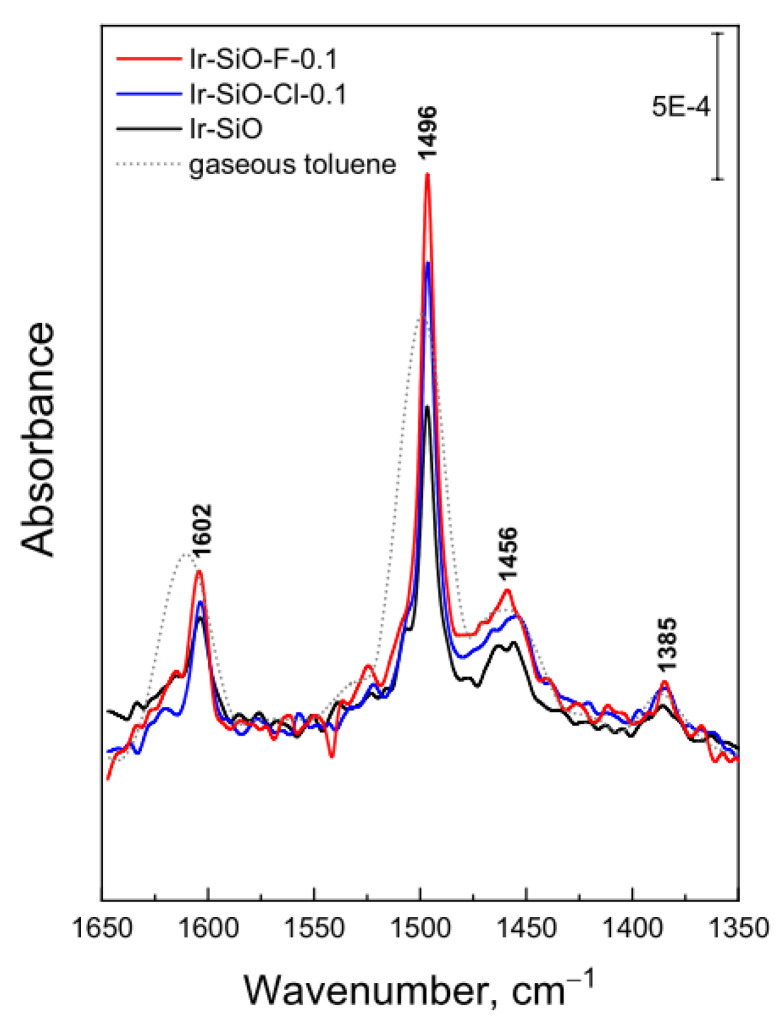
FTIR spectra of gaseous toluene and toluene adsorbed on the surface of iridium catalysts.

Figure 11 shows the spectra of gaseous toluene and toluene adsorbed on the iridium catalysts surface. The bands at 1602 and 1496 cm^−1^ are ascribed to the in-plane skeletal vibration of the aromatic ring. The band at 1385 cm^−1^ corresponds to the asymmetric deformation of the C–H bonding of the methyl group in the toluene molecule whereas the band at 1456 cm^−1^ is assigned to the stretching of the aromatic ring [45,46,47]. Chang and Kokes [48] have observed that the absorption bands at 1610 and 1500 cm^−1^ due to the gaseous toluene shifted to lower wavenumber (1593 and 1487 cm^−1^, respectively) by adsorption on zinc oxide surface. Serra et al. observed the shift of the absorption bands of toluene towards a lower wavenumber on Cs-exchanged mordenites if compared to the corresponding gas phase toluene bands [49]. According to Lian Su et al. [50], the cause of this shift are changes in electronic distribution and the symmetry of the aromatic ring as a result of toluene interactions with the zeolite structure. On the basis of the cited literature as well as the results presented in Figure 11, it can be concluded that TL is adsorbed through the formation of a π-complex on surface of investigated catalysts. The intensity of the bands increased with acidity of the support in order Ir-SiO < Ir-SiO-Cl-0.1 < Ir-SiO-F-0.1. This order correlates with the observed increase in Lewis acidity determined by pyridine adsorption and NH_3_-TPD measurements. This fact confirms the hypothesis that toluene interacts with the support surface by binding to a Lewis acid site and can be easily hydrogenated by hydrogen adsorbed on the iridium active sites.

## 4. Conclusions

In this work, the influence of acidic and textural properties of modified silica support on the activity of iridium catalysts in the hydrogenation of toluene to methylcyclohexane was determined. It was shown that the type of modifying agent used (NH_4_F or NH_4_Cl), as well as its concentration (0.1 or 1.0 M), had a significant influence on the textural and acid properties of the final supports. The modification with NH_4_F and NH_4_Cl solutions with concentrations of 0.1 M allows to obtain mesoporous supports with a surface area comparable to the original silica, and their modification leads to the generation on their surface of the Lewis type acid centers of weak and medium strength. The support modified with ammonium fluoride solution (0.1 M) was characterized by a greater proportion of acid centers as well as a larger pore size compared to the systems modified with ammonium chloride. The use of higher concentrations of modifiers (1.0 M) led to a large decrease in the BET surface area. The use of modified silica as iridium supports allowed to obtain a new class of catalysts characterized by high activities in the hydrogenation of toluene, greater than that of the iridium system supported on unmodified SiO_2_. A comparison of the activity obtained for the best Ir-SiO-F-0.1 catalyst with the results reported in the literature for iridium supported on modified acidic silica [8], alumina [26], as well as SBA-3 or SBA-15 [39], shows that the iridium catalyst supported on modified silica is more promising catalyst.

The hydrogenation activity of catalysts correlated with the acidity of the catalyst supports that influenced not only the size of the iridium particles, but also the efficiency of toluene adsorption. The size of the iridium particles decreased while the amount of adsorbed toluene increased with the acidity of the support in order Ir-SiO; Ir-SiO-Cl-0.1; Ir-SiO-F-0.1. This influenced the hydrogenation properties of the final systems. The highest activity was obtained in the presence of the catalyst supported on silica pretreated with an aqueous NH_4_F solution characterized by a higher contribution of acid sites and smaller particle size compared to other silica supports investigated.

## Figures and Tables

**Table 1:** Physicochemical properties of the unmodified and modified silicas.

Sample Code	Physical Characterization of Supports					
	Activation Conditions	S_BET_, m^2^∙g^−1^	Total Pore Volume, cm^3^∙g^−1^	Average Pore Diameter, nm	Acid Centers ^(a)^, μmol·g^−1^	Density of Acid Sites ^(a)^, µmol∙m^−2^
SiO	calcination (air, 3 h, 550 °C)	311	0.23	3.0	73.5	0.24
SiO-Cl-0.1		291	0.24	3.1	154.7	0.53
SiO-Cl-1.0		183	0.34	6.5	218.7	1.19
SiO-F-0.1		263	0.25	3.5	169.3	0.64
SiO-F-1.0		117	0.41	13.0	291.9	2.49

^(a)^ Concentration and density of acid sites were determined on the basis of NH_3_-TPD experiments.

**Table 2:** Physicochemical properties of iridium catalysts.

Sample Code	Physicochemical Characterization of Iridium Catalysts				
	Activation Conditions	S_BET_, m^2^∙g^−1^	Total Pore Volume, cm^3^∙g^−1^	Average Pore Diameter, nm	Metal Content ^(a)^, %
Ir-SiO	reduction (H_2_, 2 h, 400 °C)	277	0.25	3.6	0.71
Ir-SiO-Cl-0.1		275	0.23	3.1	0.76
Ir-SiO-F-0.1		246	0.26	3.4	0.81

^(a)^ Metal content determined by XRF method.

**Table 3:** H_2_-chemisorption data and activity of indicated catalysts.

Sample Code	Hydrogen Chemisorption Data for Ir/SiO Catalysts ^(a)^					Toluene Hydrogenation ^(b)^
	Volume Adsorbed, cm^3^∙g^−1^			Dispersion, %	Average Ir Particle Size, nm	Apparent Rate at 150 °C, mol_T_∙mol_Ir_^−1^∙min^−1^
	H_t_	H_r_	H_irr_	D		
Ir-SiO	0.17	0.09	0.08	19	5.8	7.7
Ir-SiO-Cl-0.1	0.20	0.10	0.10	23	4.9	9.7
Ir-SiO-F-0.1	0.23	0.12	0.11	24	4.8	15.8

^(a)^ Dispersion determined by H_2_ chemisorption. H_t_—total adsorbed hydrogen, H_r_—reversibly adsorbed hydrogen, H_irr_—hydrogen adsorbed irreversibly, D—dispersion calculated from hydrogen adsorbed irreversibly. Mean size of iridium particles calculated based on H_irr_. ^(b)^ Catalytic activity expressed as the apparent rate (min^−1^) in moles of toluene reacted per total moles of iridium (determined by XRF measurements).

**Table 4:** Ir(0)/IrO_2_ ratio determined from XPS analysis.

Iridium Form	Ir-SiO	Ir-SiO-Cl-0.1	Ir-SiO-F-0.1
Ir(0)	36.8%	55.1%	64.3%
IrO_2_	63.2%	44.9%	35.7%
Ir(0)/IrO_2_	0.58	1.22	1.80

## Data Availability

The data presented in this study are available on request from the corresponding author.

## Supplementary Materials

The following are available online at https://www.mdpi.com/1996-1944/14/4/968/s1, Detailed experimental procedure—FTIR analysis; H_2_ chemisorption analysis and toluene hydrogenation reaction; Figure S1: A scheme of the setup for catalytic hydrogenation of toluene; Figure S2: Experimental NH_3_-TPD profiles (gray curve) from silica supports and peaks corresponding to weak (blue), medium (green) and strong (red) acid centers obtained after deconvolution of the experimental curve; Figure S3: Deconvolution of the TPR-H_2_ profiles of the dried iridium catalysts in the range of first peak; Figure S4: N_2_ adsorption/desorption isotherms (a) and pore volume distribution as a function of pore size (b) for the iridium catalysts.
