# Supplementary material for: Effect of Modification of Amorphous Silica with Ammonium Agents on the Physicochemical Properties and Hydrogenation Activity of Ir/SiO2 Catalysts
Source: Materials (Basel). 2021 Feb 18;14(4):968. doi: 10.3390/ma14040968 (PMC7923007; doi:10.3390/ma14040968)
Supplement: Supplementary file 1 [file materials-14-00968-s001.zip › materials-1085834-supplementary.pdf]

materials

MDPI

Supplementary

Effect of Modification of Amorphous Silica with Ammonium Agents on the Physicochemical Properties and Hydrogenation Activity of Ir/SiO_2 Catalysts

Monika Kot [1,2], Robert Wojcieszak [2], Ewa Janiszewska [1], Mariusz Pietrowski [1], and Michał Zieliński [1,*]

[1] Faculty of Chemistry, Adam Mickiewicz University in Poznań, Uniwersytetu Poznańskiego 8, 61-614 Poznań, Poland; monika.kot@amu.edu.pl (M.K.); eszym@amu.edu.pl (E.J.); mariop@amu.edu.pl (M.P.)
[2] Univ. Lille, CNRS, Centrale Lille, Univ. Artois, UMR 8181-UCCS-Unité de Catalyse et Chimie du Solide, F-59000 Lille, France; robert.wojcieszak@univ-lille.fr
* Correspondence: mardok@amu.edu.pl

Detailed Experimental Procedure:

FTIR Analysis

The sample (~10 mg) was pressed into self-supporting wafer, having a surface area of 1.77 cm^2 on each face, and mounted into the IR cell equipped with KRS-5 windows. The spectra were recorded with 32 scans at 4 cm^{-1} resolution. A reference spectrum of the catalyst wafer, taken at measurement temperature, was subtracted from each spectrum. Prior to each experiment, samples were first preheated at 275 °C under reduced pressure (0.4 Pa), followed by cooling to 50 °C (pyridine adsorption) or room temperature (toluene adsorption) under dynamic vacuum conditions.

H_2 Chemisorption Analysis

The samples were evacuated for 10 min at room temperature, then at 360 °C for 30 min, followed by reduction in hydrogen flow (40 $cm^3 \cdot min^{-1}$) at 360 °C for 30 min and evacuation at 360 °C for 60 min. The additional treatment in the flow of hydrogen was aimed to clean the sample from impurities that could adsorb on the sample surface during its contact with atmospheric air. Chemisorption of hydrogen was carried out at 35 °C and the isotherms were determined using five different pressures in the range of 12–40 kPa. By assuming the stoichiometry of one hydrogen atom per one surface iridium atom (Ir_s), the dispersion of iridium (D) can be expressed as $D = Ir_s/Ir_t = H/Ir_t$ (where Ir_t—total number of iridium atoms).

Toluene Hydrogenation Reaction

Fresh dried catalyst (30 mg) was loaded into the reactor and reduced in situ in a flow (100 $cm^3 \cdot min^{-1}$) of pure hydrogen at 400 °C for 2 h before starting the reaction. After reduction the temperature was lowered to 50 °C. The reaction mixture obtained by passing hydrogen (50 cm^3 min^{-1}) through a saturator filled with toluene (Aldrich) equilibrated at 10 °C was directed to the reactor. The concentration of toluene in the feed was stable and equal to 0.75 μmol cm^{-3}. The catalysts were heated at the rate of 20 $°C \cdot min^{-1}$ in a flow of pure hydrogen and the catalytic activities were measured at temperatures between 50 °C and 225 °C in several steps over the same catalyst. The reaction was carried out for 20 min at each temperature and the products were analyzed every 10 min using a manually sampling system. Relative molar factors for toluene and methylcyclohexane were 116 and 120, respectively [1]. These coefficients were used to recalculate peak areas into molar quantities. Toluene was dosed using six-way valve that enabled the mixture to bypass the reactor. This procedure was applied in order to estimate concentration of toluene.

The reaction products were analysed on a gas chromatograph equipped with a capillary column RESTEK MXT-1. The identification of toluene and methylcyclohexane was carried out using a TCD detector.

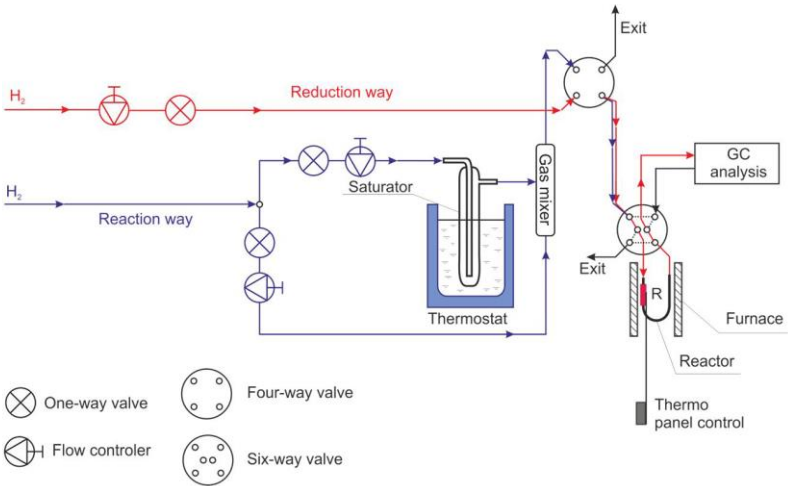

Figure S1. A scheme of the setup for catalytic hydrogenation of toluene.

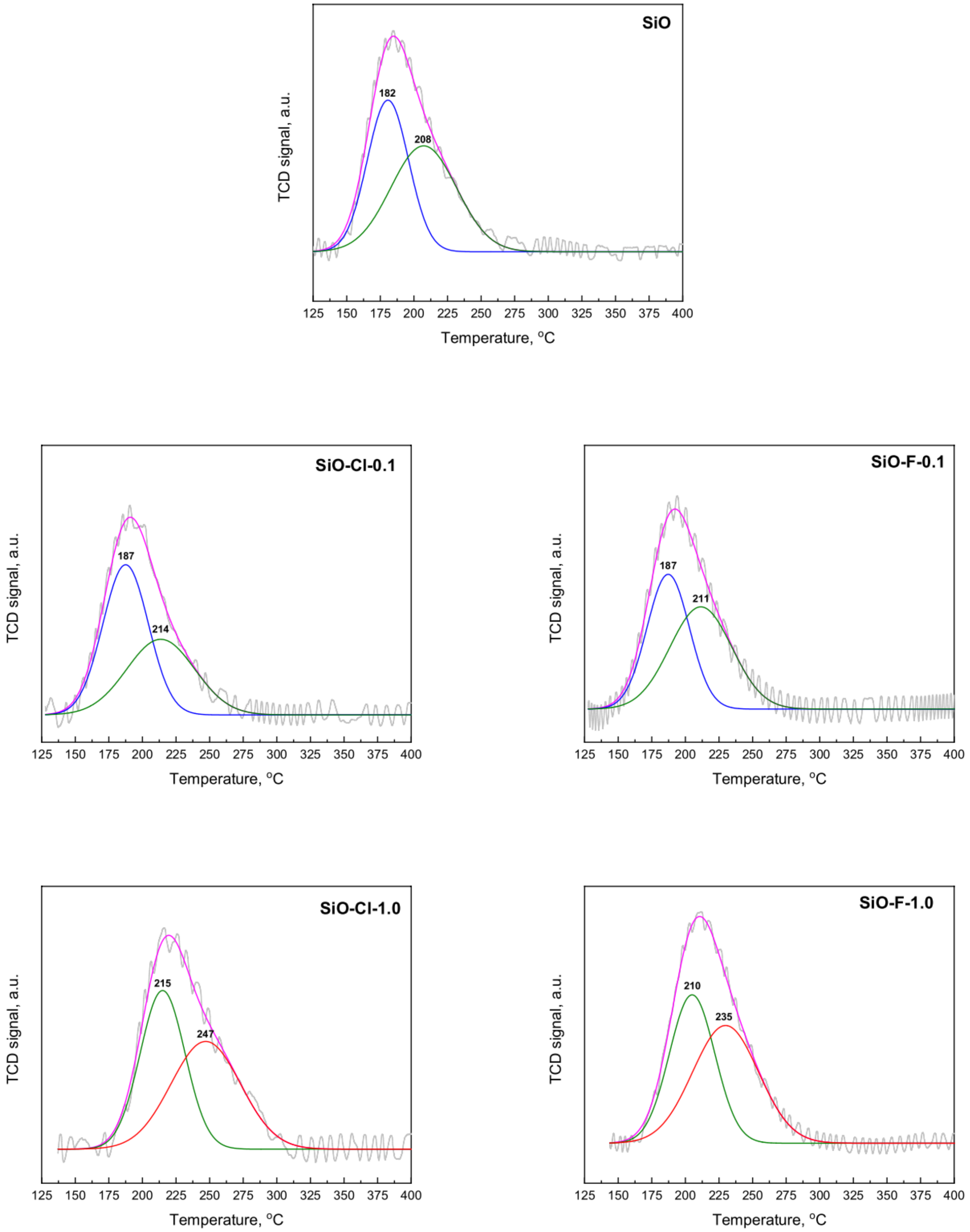

Figure S2. Experimental NH_3-TPD profiles (gray curve) from silica supports and peaks corresponding to weak (blue), medium (green), and strong (red) acid centers obtained after deconvolution of the experimental curve.

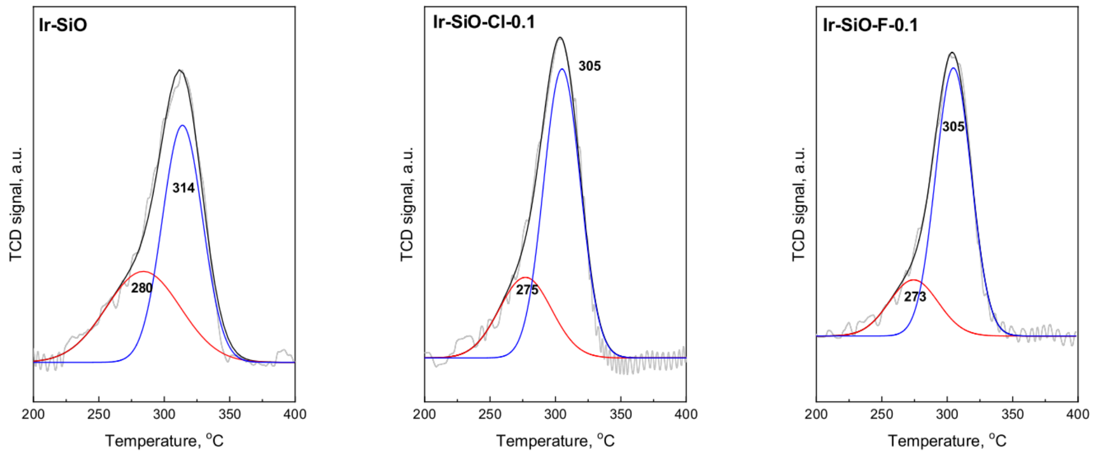

Figure S3. Deconvolution of the TPR-H_2 profiles of the dried iridium catalysts in the range of first peak.

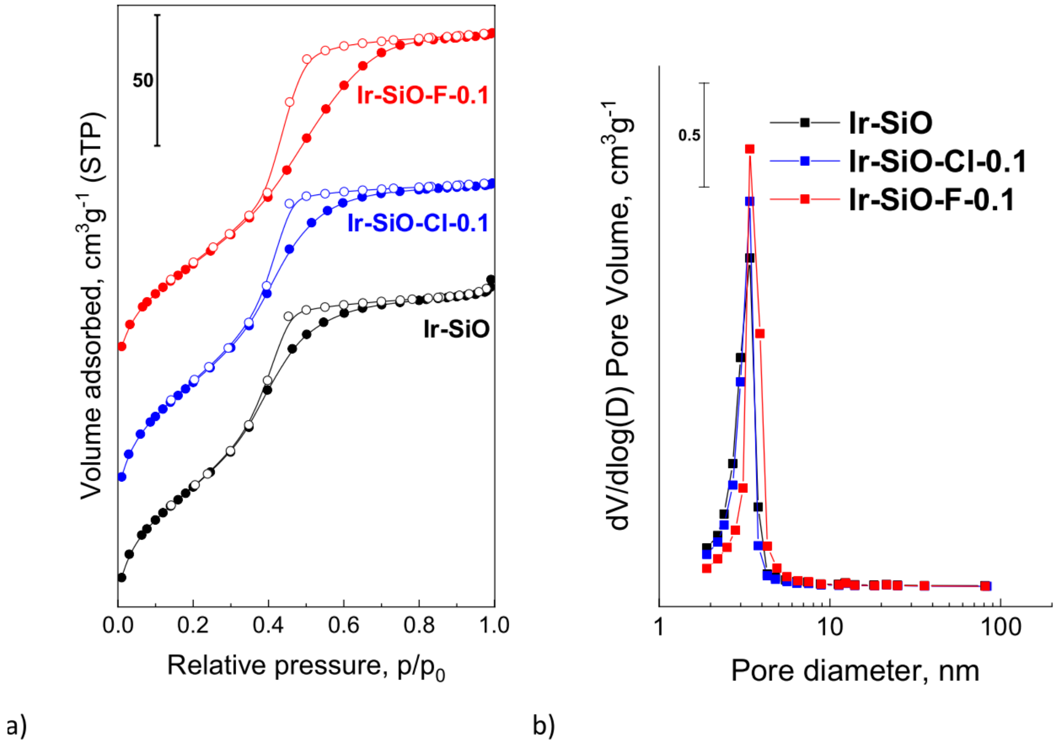

Figure S4. N_2 adsorption/desorption isotherms (**a**) and pore volume distribution as a function of pore size (**b**) for the iridium catalysts.

Reference

1. Dietz, W.A. Response Factors for Gas Chromatographic Analyses. *J. Chromatogr. Sci.* **1967**, *5*, 68–71. doi:10.1093/chromsci/5.2.68.
